# Assessment of neuropathic pain after spinal cord injury using quantitative pain drawings

**DOI:** 10.1038/s41393-021-00616-6

**Published:** 2021-02-16

**Authors:** Jan Rosner, Robin Lütolf, Pascal Hostettler, Michael Villiger, Ron Clijsen, Erich Hohenauer, Marco Barbero, Armin Curt, Michèle Hubli

**Affiliations:** 1 Spinal Cord Injury Center, Balgrist University Hospital, University of Zurich, Zurich, Switzerland; 2 Department of Neurology, Bern University Hospital, Inselspital, University of Bern, Bern, Switzerland; 3 Department of Neurology, University Hospital Zurich, University of Zurich, Zurich, Switzerland; 4 Department of Sports Medicine, Davos Hospital, Davos, Switzerland; 5 Rehabilitation Research Laboratory 2rLab, Department of Business Economics, Health and Social Care, University of Applied Sciences and Arts of Southern Switzerland, Manno/Landquart, Switzerland; 6 International University of Applied Sciences THIM, Landquart, Switzerland; 7 Department of Neurosciences and Movement Science, University of Fribourg, Fribourg, Switzerland

**Keywords:** Spinal cord diseases, Neuropathic pain

## Abstract

**Study design:**

Clinimetric cross-sectional cohort study in adults with paraplegic spinal cord injury (SCI) and neuropathic pain (NP).

**Objective:**

To assess the reliability of standardized quantitative pain drawings in patients with NP following SCI.

**Setting:**

Hospital-based research facility at the Spinal Cord Injury Center, Balgrist University Hospital, Zurich, Switzerland.

**Methods:**

Twenty individuals with chronic thoracic spinal cord injury and neuropathic pain were recruited from a national and local SCI registry. A thorough clinical examination and pain assessments were performed. Pain drawings were acquired at subsequent timepoints, 13 days (IQR 7.8–14.8) apart, in order to assess test-retest reliability.

**Results:**

The average extent [%] and intensity [NRS 0–10] of spontaneous NP were 11.3% (IQR 4.9–35.8) and 5 (IQR 3–7), respectively. Pain extent showed excellent inter-session reliability (intraclass correlation coefficient 0.96). Sensory loss quantified by light touch and pinprick sensation was associated with larger pain extent (*r*_pinprick_ = −0.47, *p* = 0.04; *r*_light touch_ = −0.64, *p* < 0.01).

**Conclusion:**

Assessing pain extent using quantitative pain drawings is readily feasible and reliable in human SCI. Relating information of sensory deficits to the presence of pain may provide distinct insights into the interaction of sensory deafferentation and the development of neuropathic pain after SCI.

## Introduction

Neuropathic pain (NP) is a frequent complication following spinal cord injuries (SCI) with a large impact on the individual’s quality of life [1–3]. Current recommendations for the assessment of NP mainly focus on uni-dimensional measures of pain, such as pain intensity, and questionnaires to capture concomitant disorders (e.g., depression, sleep disturbance) as well as the evaluation of pain interference with activities of daily living [4]. Assessments of the spatial dimension of NP using quantitative pain drawings are usually not part of these recommendations or the clinical routine. Comprehensive assessment techniques of the somatosensory system (e.g., Quantitative Sensory Testing) that are also recommended for clinical trials on NP following SCI are often performed at a particular cutaneous spot within the most painful area, whereby the spatial extent of pain is not addressed [5, 6]. Information regarding the distribution of pain is contained in the International Spinal Cord Injury Pain Basic Data Set as eight principal areas [7] based on the classical body chart designed by Margolis [8]. While such a format of data collection may be most suitable for current databases, it does not allow for an unbiased quantification of pain extent, as the regions differ in size and are not delineated along precise anatomical landmarks. Moreover, pain extent derived from quantitative pain drawings may potentially inform our pathophysiological understanding of NP after SCI by relating clinical measures of pain (e.g., widespread pain) to central sensitization processes as previously shown for other pain conditions [9].

In SCI a thorough assessment of segmental sensory and motor deficits is part of the state-of-the-art clinical examination according to the International Standards for Neurological Classification of Spinal Cord Injury (ISNCSCI) [10]. NP after SCI is then classified with regard to the neurological level of injury (NLI), with at-level pain being confined to within the dermatome of the NLI and/or three dermatomes below the lesion level, and below-level pain beginning more caudally from three segments below the NLI [4]. Relating sensory signs from the clinical examination to the presence of pain is so far limited, as the ISNCSCI exam does not distinguish between negative or positive sensory signs (i.e., hypoalgesia or hyperalgesia, respectively) [10]. To this end, the documentation of specific signs and descriptors associated with NP on standardized body charts may improve clinical practice and facilitate patient stratification for mechanism-based treatment approaches [11, 12].

The aim of this clinimetric study was to assess the reliability of standardized quantitative pain drawings in patients with spontaneous NP following SCI.

## Methods

### Participants

The present study was approved by the local ethics board ‘Kantonale Ethikkommission Zürich’ (reference number: EK-04/2006) and was part of a larger project, which also explored changes within spinal segments above the NLI. For this reason, the study only included people with paraplegia. Individuals were recruited through the national Swiss Spinal Cord Injury Cohort Study (SwiSCI) database and a local registry at the Spinal Cord Injury Center, Balgrist University Hospital, using the following criteria: (a) thoracic lesions without conus/cauda involvement in order to exclude peripheral nerve damage, (b) pain intensity assessed on a numerical rating scale (NRS) > 4 below the NLI, (d) time since injury >1 year, and (c) no documented concomitant neurological conditions. Further exclusion criteria comprised: (1) age <18 or >75 years, (2) pregnancy, (3) history, symptoms, or signs of concomitant neurological conditions other than SCI (e.g., stroke, polyneuropathy), (4) cancer (in particular with radio-chemotherapy), (5) diabetes.

### Clinical and pain phenotyping

**Fig. 1:**
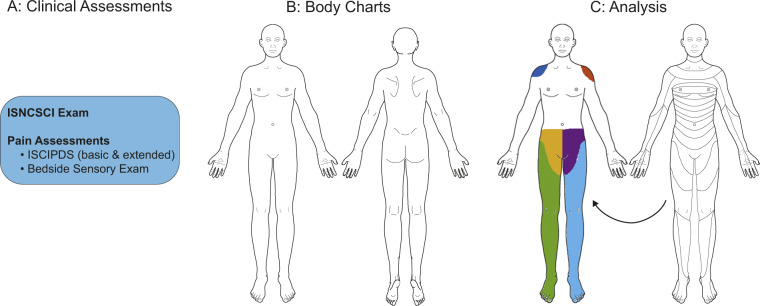
Study design. **A** Clinical and pain assessments, **B** ventral/dorsal body charts, **C** real-world example of a digitalized pain drawing of a patient with an NLI at T10. Neuropathic at-level pain is colored in yellow and violet, neuropathic below-level pain in green and light blue. Shoulder pain corresponds to the red and blue areas. A dermatome grid adapted from the ISNCSCI exam can be superimposed on the drawing in order to determine the segmental distribution of pain. ISNCSCI: International Standards for Neurological Classification of Spinal Cord Injury, ISCIPDS: International Spinal Cord Injury Pain Data Set.

A comprehensive clinical assessment (Fig. 1A) was conducted prior to the acquisition of pain drawings. The assessments included the ISNCSCI examination, and a detailed pain assessment following the recommendations of the International Spinal Cord Injury Pain Data Set (ISCIPDS) including the basic (ISCIPDS:B) and extended (ISCIPDS:E) version [13, 14]. Both questionnaires provide a thorough overview of different domains related to NP after SCI. During the examination movement-related pain was specifically asked for and sensory signs associated with NP were assessed in a bedside sensory exam focusing on the following modalities: dynamic mechanical allodynia (DMA) using a standardized brush (SENSELab Brush-05, Somedic, Sweden), pinprick hypo-/hyperalgesia using a disposable safety pin, as well as cold allodynia using a 25 °C thermoroller (Somedic, Sweden). Pinprick sensation was graded according to the ISNCSCI examination with an additional debriefing if the sensation differed from that of the control area (face). Further sensory readouts were analyzed in a qualitative manner: A decreased pinprick sensation was considered mechanical hypoalgesia, while an increased painfulness was considered mechanical hyperalgesia. Painful sensations (i.e., burning, stinging, throbbing, pulling) in response to slight brushing or the cold thermoroller were considered evidence of allodynia. After that, the pain drawings were acquired following a standardized protocol.

### Acquisition of pain drawings

Two standardized body charts (Fig. 1B) were presented to the individual on a DIN-A4 paper. The charts included a frontal and dorsal view of the body. In a standardized way, individuals were instructed to (a) familiarize themselves with the body chart including its left/right orientation, (b) shade the areas where they perceive pain, independently from type and severity of pain. Furthermore, evoked and spontaneous pain were differentiated. From the perspective of a clinimetric study evaluating a novel assessment tool, we sought to minimize variability as a result of pain fluctuations over time due to environmental, seasonal, or individual factors. For these reasons, a retest session was performed at about 2 weeks after the test session.

### Data analysis and statistics

After the examiner manually outlined the borders of the painful areas on the body charts, pain drawings were scanned and digitalized using an image analysis software (Inkscape version 0.48, GPL, USA) as described elsewhere [15]. The number of pixels per pain drawing was counted, with any shadings that extend beyond the borders of the body chart being excluded from the analysis. Pain extent was then expressed as the percentage of pixels in the frontal and dorsal view. Pain frequency maps were generated by superimposing all individual pain drawings. A dermatome grid (Fig. 1C) based on the ISNCSCI template was used to differentiate at- from below-level pain based on the standard taxonomy. The diagnosis of NP was made following current recommendations [16]. Briefly, typical sensory signs and symptoms had to be present within the painful area, and the pain distribution needed to follow a plausible neuroanatomical distribution with respect to the lesion level. Clinical judgment was used to disentangle musculoskeletal pain and NP. The classification into at- and below-level pain was performed according to the recommendations of the International Association for the Study of Pain taskforce for SCI pain [4]. For the analyses relating residual sensory function below the NLI to overall pain extent, NP extent was normalized to the body area below the NLI. Accordingly, for the pinprick and light touch scores, the residual scores relative to the NLI were calculated, i.e., the sum sensory scores within the dermatomes below the NLI were used. Following this approach, associations of below-level sensory function with below-level pain extent could be explored.

Most statistical analyses and data visualization were performed in GraphPad Prism (Version 8). ICCs were calculated using SPSS (Version 25). Normality of data distribution was tested by visual inspection of data histograms and the Shapiro–Wilk test. Descriptive statistics (mean with standard deviation (SD), or median with interquartile range (IQR), depending on data distribution) were used to report cohort and pain characteristics. Spearman correlations were used to explore associations between sensory scores and pain extent. Test–retest statistics were performed for pain extent and pain intensity. Intraclass correlation coefficients (ICCs; two-way mixed models with measures of absolute agreement) and Bland–Altman analyses were used to assess reliability. ICC values were characterized as “poor” (<0.40), “fair” (0.41–0.59), “good” (0.60–0.74), and “excellent” (0.75–1.00) [17]. The Bland–Altman analysis was carried out as described elsewhere [18]. Briefly, having tested whether the mean differences of test 1–test 2 examinations were significantly different from zero, the limits of agreement, i.e., the coefficient of repeatability (mean ± 1.96 SD) was calculated and plotted.

## Results

### Demographics and pain characteristics

Twenty individuals with SCI and NP participated in the study. A detailed cohort overview including demographics and pain characteristics is provided in Table 1. Briefly, the median age was 58.5 years (IQR 53.3–62) and the median time since injury was 14.5 years (IQR 9.4–22.3). Participants presented with sensory and motor complete (AIS A, *n* = 9) and motor incomplete (AIS C/D, *n* = 11) lesions. Musculoskeletal pain was present in 50% of individuals (*n* = 10), while at-level NP could be identified in 11/20 individuals. The median duration of NP was 14 years (IQR 9–20) with an average pain intensity during the last week [NRS 0–10] of 5 (IQR 3–7). Most individuals developed NP within the first 6 months after injury (*n* = 17), nine individuals reported pain already within the first month. Two individuals had a late onset of NP (≥24 months). Whether this delayed presentation was due to a secondary neurological deterioration, e.g., syrinx formation, could not be retrieved from the available medical records. Ten out of the 20 individuals received an anti-neuropathic medication at a stable dose for at least 3 months.

**Table 1:** Demographical information and basic information on clinical pain.

Subj. Nr.	Age	Sex [f = female, m = male]	NLI	AIS grade	Etiology [1 = traumatic, 2 = non-traumatic]	Time since injury [years]	Pain onset after injury [months]	Pain duration [years]	Pain intensity [NRS 0–10]	Presence of MSK	Presence of at-level NP	Pain medication
1	52	f	T10	A	1	23.4	<6	23	3	No	Yes	–
2	55	m	T1	A	1	11.8	<12	11	3	No	No	PGB
3	35	m	T11	C	1	14.5	<6	14	4	Yes	Yes	CB, PCM, MMZ
4	53	m	T5	A	1	20.4	<6	20	3	No	No	–
5	75	f	T10	A	2	14.4	<1	15	7	Yes	Yes	PGB, GPT
6	59	m	T9	D	1	6.7	<1	7	1	Yes	Yes	–
7	58	m	T11	A	2	24.1	<1	24	6	No	Yes	ATD, CBZ
8	62	m	T11	C	1	8.8	<1	9	8	Yes	Yes	ATD, PGB, CB
9	36	m	T11	A	1	6.7	<1	7	6	No	Yes	PGB, Voltaren
10	75	m	T10	D	2	13.8	<1	14	7	Yes	Yes	NA
11	62	m	T11	A	1	36.3	<3	36	5	Yes	Yes	–
12	66	m	T3	D	1	19.1	<1	19	8	No	No	–
13	57	m	T2	A	1	3.8	<1	4	5	Yes	Yes	ATD, PGB, CB
14	61	m	T8	D	2	11	<1	11	5	Yes	No	–
15	63	m	T12	A	1	16.8	<2	17	3	No	No	–
16	62	m	T1	D	1	36.4	~24	13	4	No	No	–
17	50	m	T3	D	2	21.3	<3	21	5.5	No	No	–
18	54	m	T10	C	1	13.3	<3	13	7	Yes	Yes	PGB, PCM
19	59	m	T12	C	1	37.6	>24	11	9	Yes	No	–
20	54	f	T5	D	2	6.2	<3	6	3	No	No	AT, MMZ

*NLI* neurological level of injury, *AIS* ASIA Impairment Scale, *MSK* musculoskeletal pain, *NP* neuropathic pain, *PGB* pregabalin, *CB* cannabinoids, *PCM* paracetamol, *MMZ* metamizol, *GPT* gabapentin, *ATD* antidepressants, *AT* amitriptyline.

### Pain extent, sensory signs and symptoms

**Fig. 2:**
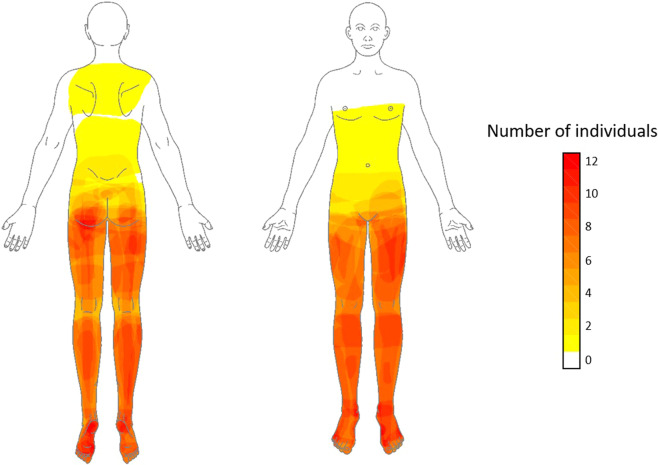
Average NP distribution across all individuals (*n* = 20). The color bar represents the frequency of NP, with dark colors indicating the most frequently reported areas of pain. Pain was most often reported within the lower limbs corresponding to the L2–L5 and S1 dermatomes.

The average NP extent was 11.3% (IQR 4.9–35.8), and musculoskeletal pain extent was 0.9 ± 1.8%. Dynamical mechanical allodynia was present in 10% of individuals and associated with at-level NP. At-level cold allodynia was present in 10% of individuals. Pain drawings of all 20 individuals were superimposed to create spatial frequency maps of the NP distribution (Fig. 2). There was no significant correlation between extent and intensity of NP (*r* = 0.33, *p* = 0.15).

### Test–retest reliability of pain extent and intensity

**Fig. 3:**
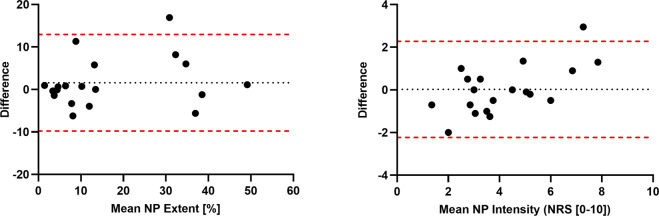
Bland–Altman plots showing the absolute test–retest reproducibility of NP extent (left) and the NP intensity (right). *NP* neuropathic pain.

The mean time interval between the test and retest session was 13 days (IQR 7.8–14.8). The test–retest reliability measures of NP extent and intensity are shown in Table 2, and the Bland–Altman plots are presented in Fig. 3.

**Table 2:** Reliability coefficients and Bland–Altman readouts for NP extent and intensity.

Test–retest reliability measures of NP extent and intensity					
	ICC	95% CI	*p* value	Bias	LoA
NP extent [%]	0.96	0.76–0.96	<0.001	1.59	−9.78 to 12.97
NP intensity (NRS [0–10])	0.91	0.90–0.99	<0.001	0.02	−2.23 to 2.27

The intraclass correlation coefficient values (ICCs) are characterized as follows: “poor” < 0.40, “fair” = 0.41–0.59, “good” = 0.60–0.74, and “excellent” = 0.75–1.00, Bias = mean difference between the two measurements.

*LoA* limit of agreement, *NRS* numeric rating scale, *NP* neuropathic pain, *CI* confidence interval.

ICCs showed an excellent reliability for pain extent (0.96) and intensity (0.91) with a narrow confidence interval. The biases for pain extent and intensity between the two testing days were very low, being less than 1% of the mean NP extent and intensity, respectively. In addition, the values of NP extent and intensity for all participants, except for one, lie within the limits of agreement.

### Sensory function and pain extent

**Fig. 4:**
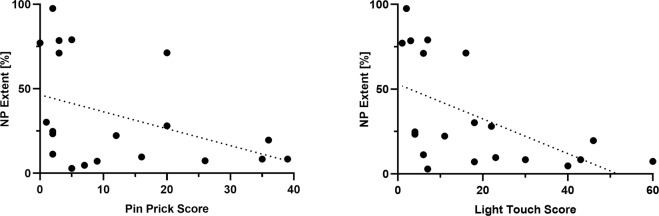
Scatter plots showing correlations of pain extent and residual sensory scores. **Left** Correlation of NP extent [%] normalized to the NLI with residual pinprick sensation below the NLI (*r* = −0.47, *p* = 0.04). **Right** Correlation of pain extent [%] normalized to the NLI with residual light touch sensation below the NLI (*r* = −0.64, *p* < 0.01). Dotted line = linear fit.

Normalized NP extent was significantly associated with sensory loss of light touch (*r* = −0.64, *p* < 0.01) and pinprick (*r* = −0.47, *p* = 0.04) below the NLI. Figure 4 illustrates that larger pain extent was correlated with more impairments of pinprick and light touch scores, respectively.

## Discussion

In the present study a novel assessment tool to quantify pain extent after SCI is presented. Our data supports the reliability of standardized, quantitative pain drawings in the assessment of NP following SCI. The quantification of pain extent emerged as highly reliable and potentially provides additional, clinically relevant information relating the presence of pain to (segmental) sensory deficits. Interestingly, pain extent was negatively correlated with residual sensory function below the NLI, potentially providing important insights into underlying pathophysiological mechanisms.

The reliability of quantitative pain drawings has been previously assessed for musculoskeletal pain syndromes presenting with widespread, referred pain [15]. For these conditions, test–retest reliability was assessed using a novel method based on a digital device [15]. The reliability of self-reported pain extent and location was excellent [15]. Our approach using a similar method of analysis showed equally excellent results. For practical reasons, we used a paper-based version of the pain drawings, however, as previously reported, completing a digital drawing or a paper-based one yields comparable results [19]. The Bland–Altman analyses showed relatively even scattering within reasonable limits of agreement for NP extent, suggesting the absence of consistent bias at either timepoints. For NP intensity, on the other hand, there seemed to be an increasing measurement variability (i.e., poorer reproducibility) at the higher and lower end of the NRS. Intra-individual fluctuations of pain intensity were previously shown to be higher for low pain ratings [20], while fluctuations associated with higher ratings could possibly indicate temporary exacerbations, e.g., pain attacks.

The analysis of pain overlap (i.e., frequency maps) showed that the pain extent clustered within the lower lumbar segments. This type of analysis will become particularly interesting when tracking an individual’s pain extent longitudinally or as an overview of the main pain distribution within a specific pain cohort. Having tools like those described in the present study, which can be readily implemented into clinical practice will provide the foundation for such analyses in the future. The assessment of the neuroanatomical distribution of pain is also of crucial diagnostic importance in NP conditions. Following current recommendations, NP has to show an anatomically plausible distribution [16], which can be inferred from individual pain drawings. As pain following SCI is classified with regard to the NLI into at-, below- and above-level pain [21], information regarding location is also of particular interest in NP following SCI.

While the pain extent derived from standardized pain drawings informs the clinician about the neuroanatomical distribution of pain [22], it may also be indicative of underlying pathophysiological processes, e.g., widespread pain as a clinical correlate of central sensitization processes [23, 24]. For instance in musculoskeletal pain conditions, pain extent was shown to be associated with more severe pain reflected in higher ratings on the NRS or with several direct and indirect measures related to central sensitization, e.g., painDETECT scores or pressure pain thresholds [23]. These findings have been discussed in the context of impaired endogenous pain modulation and sensitization processes (for review see [25]). Moreover, in a study on chronic pelvic pain, measures of pain extent were related to cerebral reorganization and interpreted in the framework of “pain centralization”, implying that pain is maintained predominantly by central processes, detached from peripheral input [9]. Interestingly, as opposed to musculoskeletal pain conditions, there was no significant correlation of pain extent with pain intensity in our cohort of individuals with central NP after SCI. A possible explanation for this could be related to differences in the underlying pathophysiology of the two conditions. Whereas musculoskeletal pain is driven by enhanced peripheral input which then initiates and maintains central sensitization [25], NP after SCI may be more related to spinal and supraspinal maladaptive plasticity, which, to an indeterminate extent, may be the consequence of deafferentation [26, 27]. Thus, in the case of musculoskeletal pain, the spatial extent of pain may be the result of increased responsiveness in nociceptive neurons (i.e., central sensitization) [23] and is therefore also accompanied by an increased pain intensity. In NP after SCI, on the other hand, pain may be primarily discoverable as complex structural and functional changes rostral to the spinal lesion [28], which may render the relationship between pain intensity and pain extent less direct.

Relating changes in sensory function to specific spinal segments is fundamental in the clinical examination of patients with SCI and is therefore implemented in the ISNCSCI exam [10]. However, the exam falls short of documenting sensory plus signs like allodynia and hyperalgesia, which may precede the onset of spontaneous NP after SCI [29]. Standardized pain drawings combined with bedside sensory testing may close this important diagnostic gap. Sensory deficits or heightened sensitivity can be specifically assessed within the painful area or within adjacent segments. In our cohort, we revealed sensory plus signs (i.e., dynamical mechanical allodynia and cold allodynia) confined to the areas of at-level pain in a subset of individuals. Such information may become important in the longitudinal assessment of patients enrolled in clinical trials aimed at enhancing neuroplasticity. Here, the emergence of NP as a complication is a relevant concern and sensitive assessment tools are lacking so far [30]. Segmental assessments of pain and sensory dysfunction are of paramount importance in order to be able to relate the presence of pain to the clinical-neurological status, and thereby immediately detect subtle segmental deteriorations. In trials related to the treatment of NP, pain extent and segmental involvement relative to the lesion level could be promising outcome parameters as they may help to differentiate treatment effects related to peripheral (i.e., effect on at-level pain) or central components (i.e., effects on below-level pain) of the NP phenotype.

The pathophysiological mechanisms that give rise to NP after an injury to the spinal cord are complex and incompletely understood [31, 32]. Our data corroborate the notion of deafferentation-related pain as pain extent was negatively correlated with preserved sensory function of both medial-lemniscal (i.e., light touch) and spinothalamic (i.e., pinprick) modalities below the NLI. A pathological disruption of spinal cord integrity is associated with regional changes around the lesion site ranging from inflammatory reactions, excitotoxicity, and glial cell activation eventually leading to altered neuronal excitability and possibly behavioral hypersensitivity [32]. Moreover, anterograde and retrograde degeneration of ascending or descending projections may result in impaired endogenous control of afferent nociceptive input as well as neuronal deafferentation of sensory relay areas rostral to the spinal cord lesion [33, 34] Damage to peripheral afferent fibers (e.g., compromised nerve roots in at-level NP) with ensuing ectopic activity may also contribute to the spontaneous pain phenotype [35]. Such a contribution of peripheral afferent input to central NP is a timely topic, and was recently proposed as a contributing mechanism in central post-stroke pain [36].

The present study highlights the reliability of quantitative pain drawings in the assessment of NP after SCI. Mechanistically, the relationship between sensory loss and pain extent reinforces the pathophysiological concept of deafferentation-related NP. Future studies are warranted to explore the relationship of markers related to central sensitization and pain extent in SCI. Overall, the method of quantitative pain drawings can be readily implemented into the clinical routine and potentially provides added clinical and pathophysiological information, with the prospect of becoming an outcome measure in SCI clinical trials.

## Limitations

A notable strength of this study is the rigorous exclusion of concurrent peripheral nerve damage and comorbidities that may confound the primary outcome, i.e., the extent of central NP. Although specifically addressed during the initial interview, the presence of musculoskeletal pain below the neurological level of injury cannot be conclusively ruled out and may contribute to the clinical pain phenotype. Such a remaining uncertainty is a “common situation in neurological diagnostics” [16] and there is currently no hard-and-fast algorithm on how to disentangle neuropathic from musculoskeletal pain below the neurological level of injury.

## Data Availability

The datasets generated and/or analyzed during the current study are available from the corresponding author on reasonable request.
